# Retinol dehydrogenase-10 promotes development and progression of human glioma via the TWEAK-NF-κB axis

**DOI:** 10.18632/oncotarget.22166

**Published:** 2017-10-27

**Authors:** Feng Guan, Liang Wang, Shuyu Hao, Zhen Wu, Jian Bai, Zhuang Kang, Quan Zhou, Hong Chang, Hui Yin, Da Li, Kaibin Tian, Junpeng Ma, Guijun Zhang, Junting Zhang

**Affiliations:** ^1^ Department of Neurosurgery, Beijing Tiantan Hospital, Capital Medical University, Beijing, China; ^2^ CAS Key Laboratory of Genome Sciences and Information, Beijing Institute of Genomics, Chinese Academy of Sciences, Beijing, China; ^3^ Department of Glioma, Beijing Shijitan Hospital, Capital Medical University, Beijing, China; ^4^ Department of Pathology, Beijing Shijitan Hospital, Capital Medical University, Beijing, China; ^5^ Department of Social Medicine and Health Education, School of Public Health, Peking University, Beijing, China

**Keywords:** retinol dehydrogenase 10 (RDH10), glioma, the cancer genome atlas (TCGA), tumor necrosis factor-like weak inducer of apoptosis (TWEAK), nuclear factor kapaB (NF-κB)

## Abstract

Retinol dehydrogenase-10 (RDH10) is a member of the short-chain dehydrogenase/reductase family, which plays an important role in retinoic acid (RA) synthesis. Here, we show that RDH10 is highly expressed in human gliomas, and its expression correlates with tumor grade and patient survival times. *In vitro*, lentivirus-mediated shRNA knockdown of RDH10 suppressed glioma cell proliferation, survival, and invasiveness and cell cycle progression. *In vivo*, RDH10 knockdown reduced glioma growth in nude mice. Microarray analysis revealed that RDH10 silencing reduces expression of TNFRSF12A (Fn14), TNFSF12 (TWEAK), TRAF3, IKBKB (IKK-β), and BMPR2, while it increases expression of TRAF1, NFKBIA (IκBα), NFKBIE (IκBε), and TNFAIP3. This suggests that RDH10 promotes glioma cell proliferation and survival by regulating the TWEAK-NF-κB axis, and that it could potentially serve as a novel target for human glioma treatment.

## INTRODUCTION

Glioma is one of the most fatal types of human brain cancers, and currently there is no effective therapy [1]. According to clinicopathological parameters, the World Health Organization (WHO) has classified human gliomas into grades I–IV [1], which are highly related to progression, treatment and prognosis. Pathological grades for glioma are usually divided into low-grade and high-grade. Low-grade gliomas (LGG) include grades I and II, which often have better prognoses, while high-grade gliomas (HGG) include grades III and IV and have worse prognoses. Malignant gliomas, one of the most aggressive human brain cancers, account for 70%-80% of malignant brain tumors [2–5]. Despite achievements in surgical procedures, radiotherapy and chemotherapy, the 5-year survival rates for gliomas remain low [6, 7]. To achieve better outcomes, a better understanding of the responsible molecular pathways and mechanisms is urgently needed.

Retinol dehydrogenase-10 (RDH10) is a member of the short-chain dehydrogenase/reductase family that was first cloned from human, mouse and bovine retinal pigment epithelial cells [8]. The homology in the amino acid sequences among RDH10 isoforms from different species is extraordinarily high, with a 99% identity between human and murine RDH10 [8], indicating a high functional significance of RDH10. Rdh10-null mice are embryonic lethal, but can be rescued by retinoic acid (RA) treatment of the pregnant mother, suggesting that RDH10 is essential for RA generation during embryonic development [9]. While the exact function of RDH10 in RA generation is unknown, it has been reported that RDH10 mediates retinol (vitamin A) oxidation to generate retinal, which is indispensable for retinoic acid synthesis [10, 11]. As retinoic acid is used in the treatment of many types of leukemia, it is possible that RDH10, an important enzyme in retinoic acid synthesis, might be involved in cancer development.

Tumor necrosis factor-like weak inducer of apoptosis (TNFSF12, TWEAK) and its receptor, fibroblast growth factor-inducible protein14 (TNFRSF12A, TWEAKR, Fn14), are involved in many biological processes, including regulation of NF-κB signaling [12–17]. NF-κB signaling controls many biological processes, including cell survival, and immune and inflammatory responses [18]. The canonical NF-κB pathway is activated by inducible phosphorylation and degradation of IκBα, resulting in the nuclear translocation of NF-kB subunits. The non-canonical NF-κB pathway is regulated by NF-κB inducing kinase (NIK) and ubiquitin-mediated proteasomal degradation of TRAF3 [19–23]. Previous studies have found that TWEAK and Fn14 are over-expressed in gliomas that have high levels of NF-κB activation [24–27], indicating that NF-κB may serve as an important therapeutic target in glioma [28].

In this study, we have analyzed the RDH10 expression in human gliomas, and investigated the role of TWEAK–NF-κB axis during glioma development. Our results demonstrate that RDH10 is highly expressed in gliomas and correlates with poor prognosis, suggesting that it may serve as a new target for glioma treatment.

## RESULTS

### RDH10 expression is associated with development and progression of human glioma

**Figure 1:**
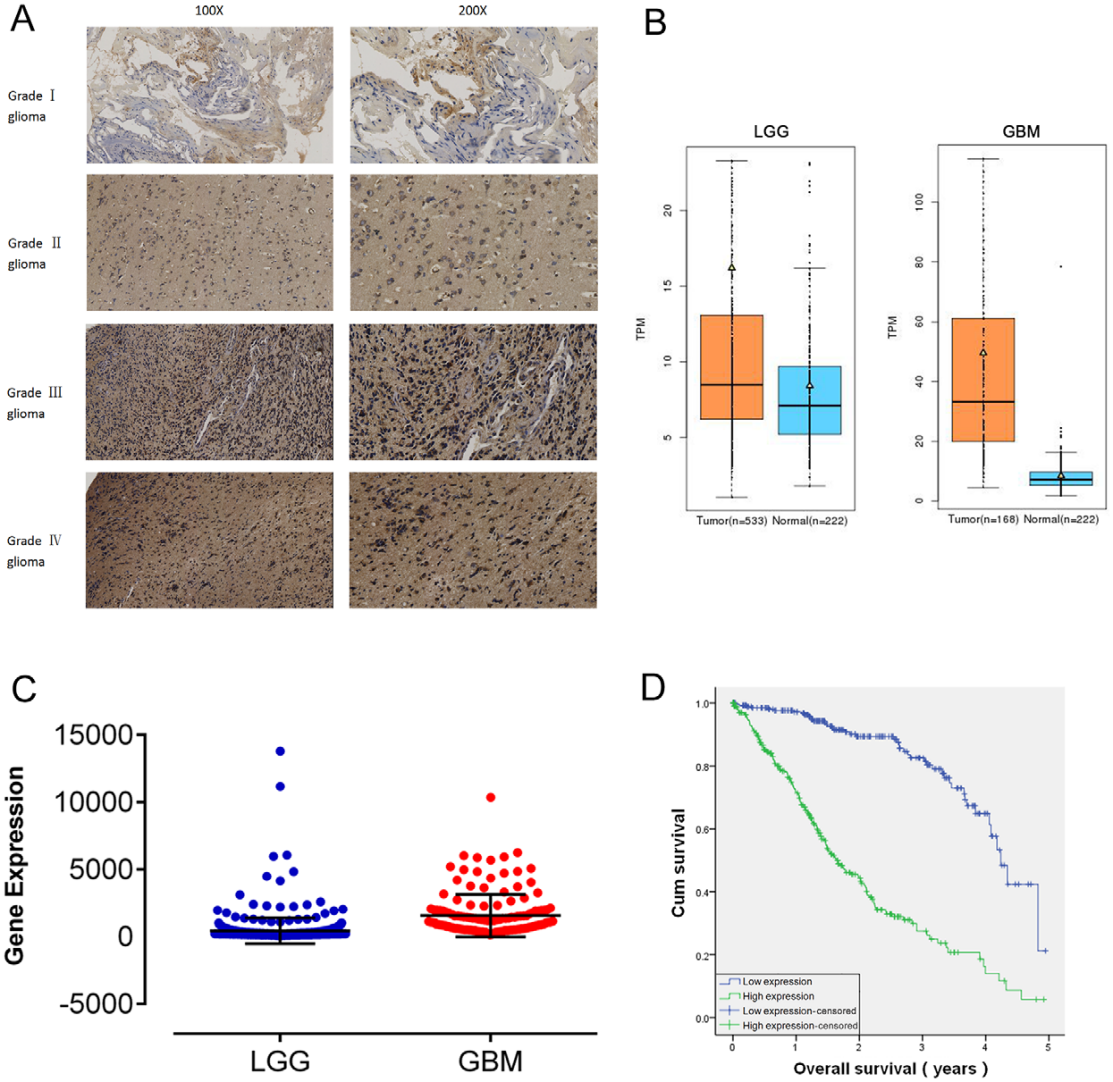
RDH10 is over-expressed in human gliomas and predicts a high grade and poor prognosis **(A)** Characteristic micrographs of RDH10 immunostaining in gliomas at WHO grade I, II, III, and IV (magnification left, ×100 and right, ×200). **(B)** Analysis of RDH10 expression from GE-mini(TCGA and GTEx datasets) revealed significant differences between normal brain tissues and glioma. **(C)** Analysis of RDH10 expression in LGG and GBM. A generalized linear model (GLM) analysis was performed for TCGA RNA-sequencing data of Gliomas. ^**^P < 0.01. **(D)** Survival analysis showed that higher RDH10 expressing gliomas had a poorer prognosis than lower RDH10 expressing gliomas. ^**^P < 0.01.

In our study, 150 human glioma samples of grades I–IV were harvested to evaluate RDH10 expression by immunohistochemistry (IHC) (Figure 1A). IHC data demonstrated that RDH10 was expressed in glioma specimens of different grades to different degrees. Positive RDH10 expression was found in 5/36 grade I glioma specimens, 17/39 grade II specimens, 24/34 grade III specimens, and 37/41 grade IV specimens (Table 1). These data demonstrate that RDH10 expression positively correlates with the pathological grade in human glioma (*P*<0.001). Analysis of RDH10 expression from GE-mini dataset (http://gemini.cancer-pku.cn/)[29], which integrates The Cancer Genome Atlas (TCGA) and Genotype-Tissue Expression (GTEx) datasets, revealed that RDH10 levels are increased in gliomas compared with normal brain tissues, and highly increased in glioblastoma (GBM) (Figure 1B). In TCGA dataset, the RDH10 expression also exhibited significant differences between LGG and GBM (^**^P< 0.01) (Figure 1C, Supplymentary Table 1). A generalized linear model analysis was then performed on TCGA RNA-sequencing data for gliomas, and Kaplan-Meier survival curve was used to evaluate patient survival times. These results showed that the cumulative survival rate was remarkably lower in glioma patients with higher RDH10 expression than in those with lower RDH10 expression (^**^P < 0.01) (Table 2, Figure 1D). As both gliomas grade and RDH10 expression had a significant influence on patient survival, we wondered whether RDH10 expression was an independent prognostic factor of glioma patients’ survival. Analysis with Cox's regression model revealed that RDH10 expression had independent impact on glioma patients’ survival (Table 3, B=1.068, ^***^P < 0.001). As the coefficients of Cox's regression model was positive (B value=1.068), these data suggested that the higher RDH10 level indicated a worse prognosis for glioma patients, and was associated with a 2.908-fold increased risk of death (Exp(B)=2.908). Together, these data indicate that the RDH10 expression may serve as a potential biomarker in the pathogenesis and progression of gliomas.

**Table 1:** Association between RDH10 expression and WHO grading of gliomas

WHO grading	*N*	RDH10 expression	
		Positive(n)	%
Grade I	36	5	13.89
Grade II	39	17	43.59
Grade III	34	24	70.59
Grade IV	41	37	90.24

**Table 2:** Correlation between RDH10 expression and patient survive in TCGA

		Patient	Death	Death	*P* value
		N	N	%	
RDH10 expression level	Low	286	45	15.7	0.000
	High	311	171	55	
	Total	597	216	36.2	

**Table 3:** Cox regression analysis results

	B	P value	Exp (B)	95.0% CI of Exp(B)	
				Lower	Upper
RDH10 level	1.068	.000	2.908	1.976	4.280

### Lentiviral-mediated shRNA efficiently inhibits RDH10 expression

**Figure 2:**
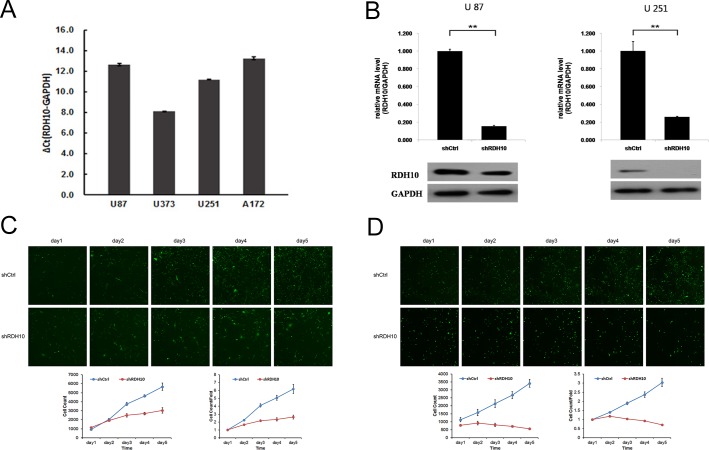
Lentiviral-mediated RDH10 knockdown suppressed human glioma proliferation **(A)** Relative RDH10 mRNA levels in glioma cell lines by qPCR. **(B)** qPCR revealed that RDH10 expression was efficiently reduced in U87 and U251 cells. ^**^P < 0.01. **(C and D)** RDH10 knockdown inhibited U87 (C) and U251 (D) proliferation *in vitro*. Proliferation detected by CellomicsArrayScan VTI every day for 5d. All experiments were repeated at least three times. Data are shown as mean ± SD, ^**^P < 0.01.

To investigate whether there is a causal relationship between RDH10 expression and development of human glioma, we examined the RDH10 expression in glioma cell lines U87, U251, U373 and A172. Gene expression of RDH10 was moderate in U87 and A172 cells, and high in U373 and U251 cells (Figure 2A). To establish glioma cell lines with suppressed RDH10 expression, glioma cells were treated with RDH10-shRNA or scrambled (Scr)-shRNA viruses and RDH10 gene and protein levels were analyzed by real-time PCR (qPCR) and western blotting, respectively. Gene and protein levels of RDH10 were significantly decreased in cells that received RDH10-shRNA (Figure 2B), demonstrating that the lentivirus-based shRNA strategy efficiently inhibited RDH10 expression in glioma cells.

### RDH10 knockdown impairs glioma cell proliferation *in vitro*

**Figure 3:**
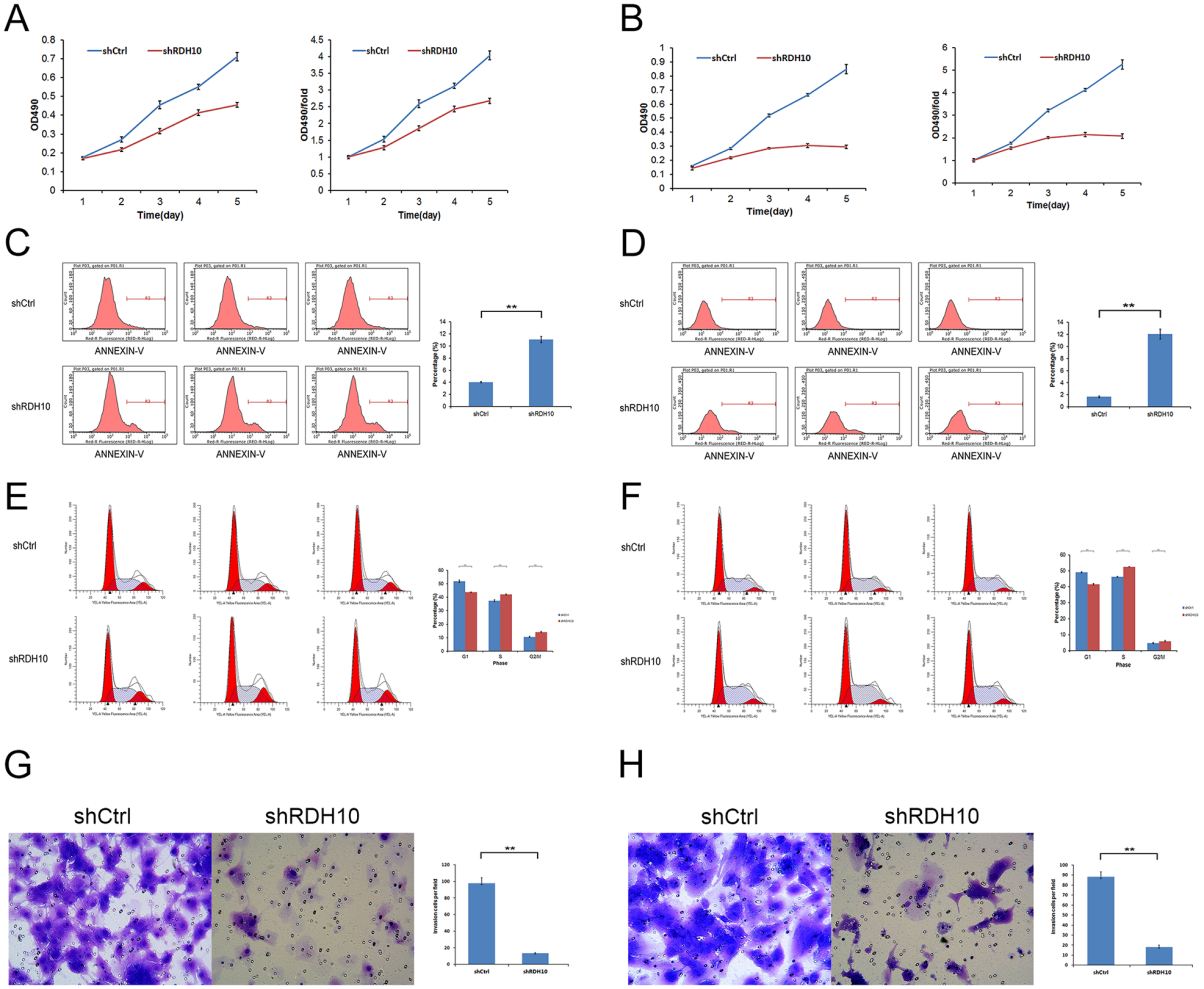
RDH10 is essential for glioma cell proliferation, survival and invasion **(A and B)** MTT assays indicated that the growth rates decreased in RDH10-silenced U87 (A) and U251 (B) cells. **(C and D)** RDH10 knockdown induced apoptosis in U87 (C) and U251 (D) cells. **(E and F)** RDH10 knock- down induces an S and G2/M cell cycle arrest in U87 (E) and U251 (F) cells. **(G and H)** Transwell Matrigel invasion assays showed that the number of invasive cells in the RDH10 siRNA group was decreased significantly compared with that in the control group for U87 (G) and U251 (H) cells. All experiments were repeated at least three times. Data are shown as mean ± SD, ^**^P < 0.01.

Persistent proliferation signals are an important marker of cancer [30]; thus, it was important to study the impact of RDH10 knockdown on glioma cell proliferation. We assessed cell proliferation with two different assays to reduce subjective factors. First, cell proliferation was monitored using CellomicsArrayScan VTI for five consecutive days (Figure 2C and 2D). These results showed a distinct reduction in cell proliferation starting 48 h after the cells were treated with RDH10-shRNA compared with Scr-shRNA–treated cells. Moreover, the restrictive impact of RDH10-shRNA on proliferation increased over time (Figure 2C and 2D). Second, cell proliferation was assessed by the MTT assay, which showed that the growth rate of RDH10-knockdown cells was lower than Scr-shRNA cells 3, 4 and 5 d after plating (^**^P< 0.01) (Figure 3A and 3B).

### RDH10 knockdown promotes glioma apoptosis

Resistance to apoptosis is another important feature of cancer cells [31–33]. RDH10 knockdown induced a reduction in cell numbers (Figure 2C and 2D) that was likely due to impaired cell proliferation and/or increased apoptosis. Therefore, we used flow cytometry and Annexin V-APC to assess apoptosis in U87 and U251 cells treated with RDH10-shRNA. The percentage of apoptotic U87 cells was significantly enhanced in the RDH10-shRNA group compared with the Scr-shRNA group (11.06 ± 1.41 % vs 4.03 ± 0.09%, respectively; *P* = 0.013) (Figure 3C). The percentage of apoptotic U251 cells also significantly increased following RDH10-shRNA treatment compared with Scr-shRNA (12.04 ± 1.12 % vs 1.67 ± 0.12%, respectively; *P* = 0.0036) (Figure 3D), indicating that RDH10 may have an anti-apoptotic role in glioma cells.

### RDH10 knockdown induces glioma cell cycle arrest

To investigate the effect of RDH10 on cell cycle, propidium iodine-stained cells were analyzed by flow cytometry. For U87 cells, the Scr-shRNA group showed the distribution, G0/G1: 51.97%, S: 37.46% and G2/M: 10.58%, and the RDH10-shRNA group showed G0/G1: 43.74%, S: 42.12% and G2/M: 14.14% (Figure 3E). For U251 cells, the Scr-shRNA group showed the profile, G0/G1: 49.1%, S: 46.21% and G2/M: 4.69%, while the RDH10-shRNA group showed G0/G1: 41.6%, S: 52.58% and G2/M: 5.82% (Figure 3F). Suppression of RDH10 in both cell types reduced the number of cells in the G0/G1 phase, and increased the number of cells in the S and G2/M phases, suggesting that RDH10 regulates the cell cycle progression, as its loss causes an S and G2/M phase arrest (^**^P< 0.01, Figure 3E and 3F).

### RDH10 knockdown inhibits glioma cell invasion ability

Next, we evaluated the effect of RDH10 on cell invasion using matrigel-coated trans-well assay. RDH10-shRNA significantly inhibited cell invasion ability of U87 and U251 cells. Invaded cells of Scr-shRNA and RDH10-shRNA groups were 97 ± 6.73 and 13 ± 0.59 in U87 cells, respectively (Figure 3G). Invaded cells of Scr-shRNA and RDH10-shRNA groups were 88 ± 5.04 and 18 ± 1.81 in U251 cells, respectively (Figure 3H) (^**^P< 0.01). These results indicate that RDH10 knockdown inhibits cell invasion ability in glioma cells.

### RDH10 shRNA inhibits glioma cell growth *in vivo*

**Figure 4:**
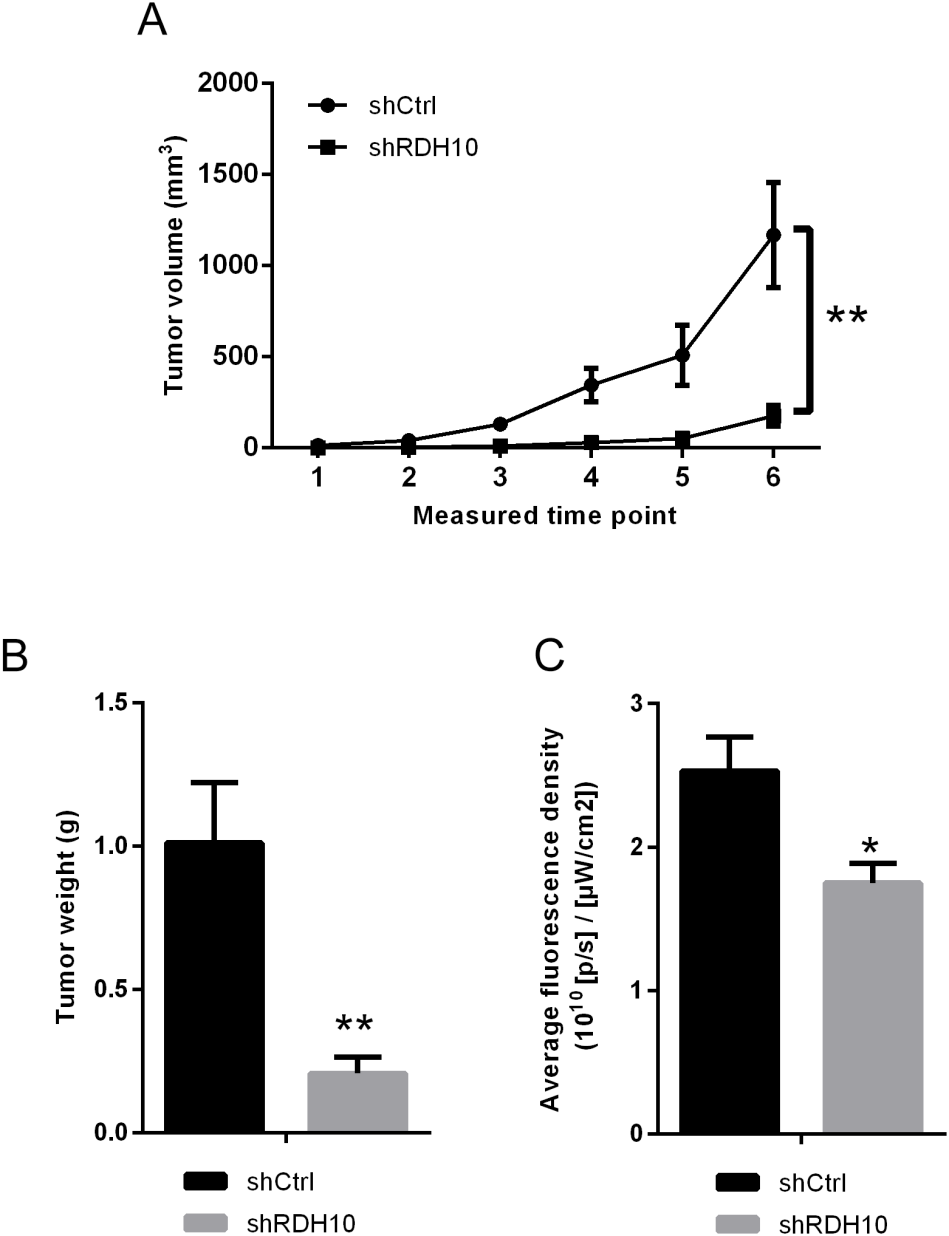
*in vivo* xenograft models confirmed the effect RDH10 on tumorigenicity **(A)** Tumor growth curves of RDH10-shRNA U87 cells compared with control lentivirus infected cells. **(B)** Tumor weights were compared at the end of the experiment. **(C)** Fluorescence density of tumors were compared at the end of the experiment. ^**^P <0.01, ^*^P =0.011.

To study whether RDH10 silencing affects the growth of glioma cells *in vivo*, U87 cells transfected with RDH10-shRNA or scrambled-shRNA were inoculated into nude mice to establish xenograft tumor model. The volumes of glioma xenografts with suppressed RDH10 were significantly smaller than the control tumors (Figure 4A). Furthermore, both tumor weight and fluorescence density were significantly lower in the RDH10-shRNA group than in the control Scr-shRNA group (Figure 4B-4C, n=10, *P*=0.0017 and 0.011, respectively). These results confirmed the *in vitro* data, and indicated that the increased expression of RDH10 induces glioma development and progression.

### RDH10 regulates expression of glioma genes

**Figure 5:**
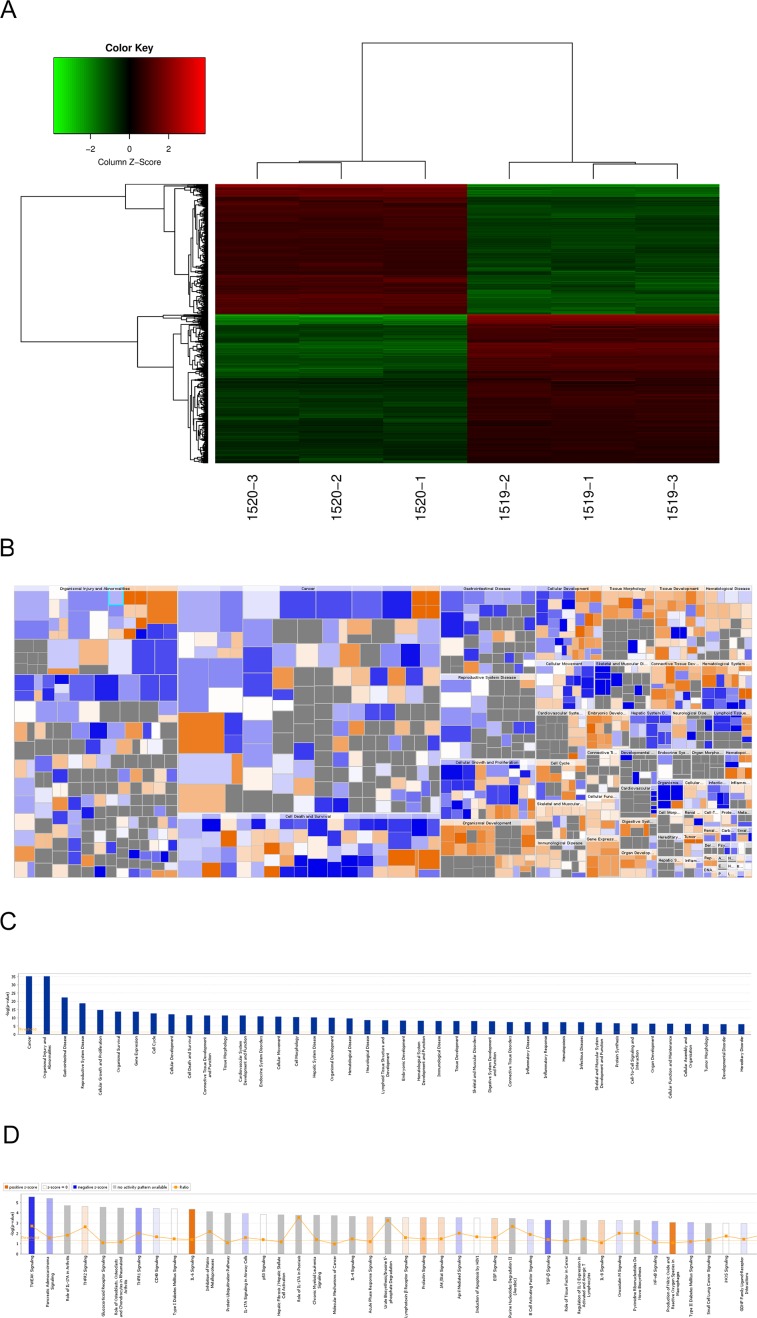
Changes in gene expressions in U-87 cells with RDH10 knockdown by microarray **(A)** Heat-map analysis showing 1773 genes that were detected as altered by microarray profiling. corrected *P*<0.05 and absolute FC >1.5. **(B and C)** Diseases and functions enrichment of whole-genome expression microarray in RDH10 knockdown U87 cells were analyzed by IPA. **(D)** Canonical pathways enrichment of whole-genome expression microarray in RDH10 knockdown U87 cells were analyzed by IPA.

To investigate the mechanisms of how RDH10 regulates glioma progression, we performed whole-genome expression microarray on U-87 cells expressing either Scr-shRNA or RDH10-shRNA. We detected 1773 genes that displayed differential expression (corrected *P*<0.05 and absolute FC≥1.5), including 850 up-regulated genes and 923 down-regulated genes (Figure 5A). According to the Ingenuity Pathway Analysis (IPA) database, RDH10 knockdown affected expression of genes involved in cancer, apoptosis, growth and proliferation, motility and cell cycle (Figure 5B and 5C). Furthermore, RDH10 knockdown significantly repressed several key cancer pathways including TWEAK, TNFR1 and P53 (Figure 5D), indicating that RDH10 regulates malignant phenotypes in human glioma.

### RDH10 knockdown inhibits glioma cell growth by down-regulating the TWEAK–NF-κB axis

**Figure 6:**
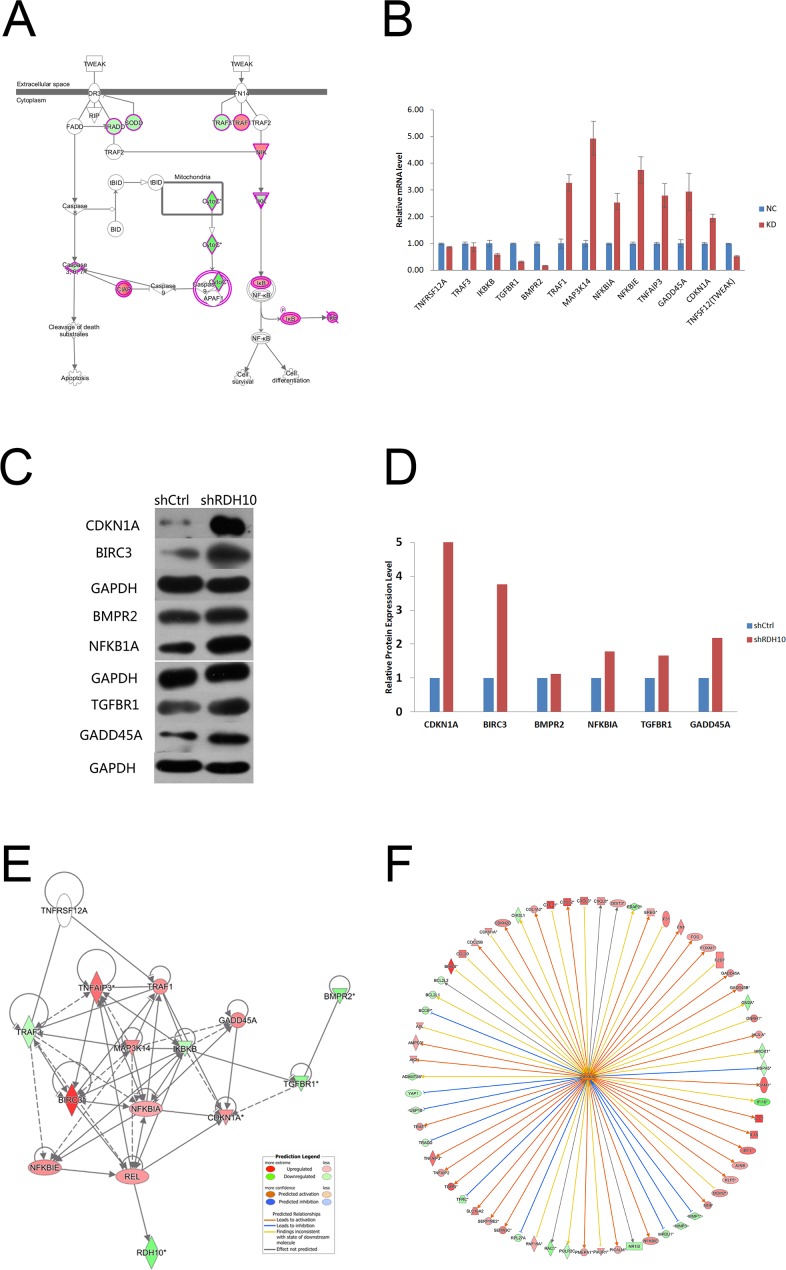
TWEAK–NF-κB signaling pathway was significantly inhibited in RDH10 knockdown U87 cells **(A)** IPA showing that TWEAK signaling was one of the most significantly inhibited pathway. **(B)** qPCR was used to validate the mRNA expression of related genes. **(C)** Western blotting was used to validate the protein expression levels of related genes. **(D)** Densitometry showed relative protein expression levels of related genes. **(E and F)** Gene interaction network of RDH10 and related genes in TWEAK–NF-κB signaling pathway. The results show that NFKBIA is located in the central regulatory position. Data are shown as mean ± SD.

**Figure 7:**
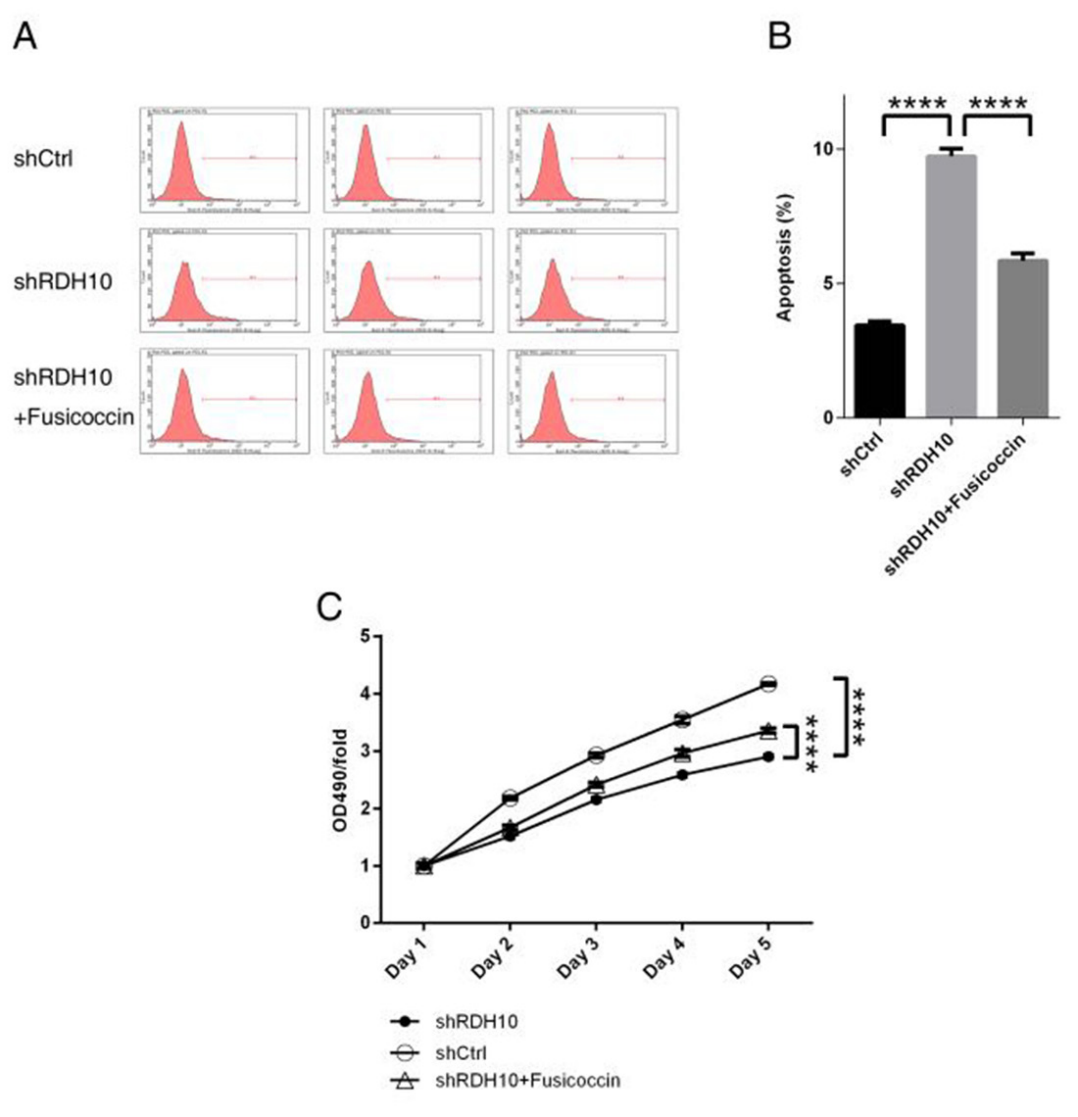
NF-κB agonist rescued abnormal cellular apoptosis and proliferation induced by RDH10 knockdown in U-87 cells **(A-B)** NF-κB agonist fusicoccin blocked up-regulated cellular apoptosis mediated by RDH10 knockdown in U87 cells. **(C)** NF-κB agonist Fusicoccin rescued impaired cellular proliferation status mediated by RDH10 knockdown in U-87 cells. cellular proliferation status was analyzed by MTT assay. ^****^P <0.0001.

Previous studies have shown that TWEAK activates NF-κB-dependent genes, including MMP9 [16, 17]. Our microarray analysis indicated that the TWEAK–NF-κB pathway was inhibited after RDH10 knockdown (Figure 5D and 6A). Further analysis of gene and protein levels by qPCR and western blotting validated the microarray data; compared with the control Scr-shRNA group, RDH10 silencing reduced expression of TNFRSF12A (TWEAKR, Fn14), TNFS12 (TWEAK), TRAF3, IKBKB (IKK-β), TGFBR1, and BMPR2, while it increased expression of TRAF1, MAP3K14 (NIK), NFKBIA (IkBα), NFKBIE (IkBε), TNFAIP3, GADD45A, and CDKN1A (Figures 6B-6D). According to the IPA database, IkBα played a central role in the regulation (Figure 6E), and was activated (Figure 6F). Expression of downstream genes involved in cancer development, such as BIRC3, BCL2L1, BCL2L2, CDKN1A, DDIT3, GADD45A, GADD45B, MMP1, MMP2, MMP3, MMP7, RAC1 and YAP1, is shown in Supplementary Table 2. Next, we used the NF-κB agonist fusicoccin to investigate the relationship between RDH10 and NF-κB. We found that increased apoptosis (Figures 7A, 7B) and impaired cellular proliferation (Figure 7C) induced by RDH10 knockdown could be partially rescued by fusicoccin, thus supporting the direct link between RDH10 and NF-κB.

## DISCUSSION

Since there are no effective therapies for malignant glioma, it is important to identify new, suitable, therapeutic targets. Only a few tumor types are associated with RDH10. Roles of RDH10 in cancer have been investigated in hepatocellular cancer cell line (HepG2), which showed that RDH10 over-expression inhibited cell growth. Reports of lung cancer showed that RDH10 mutations were common in malignant non-small-cell lung cancer [34–36]. RDH10 plays an important role in the synthesis of RA, which regulates cell proliferation and differentiation. The key molecules in RA signaling regulate biological behavior of glioma stem-like cells (GSCs), such as proliferation, invasion, and angiogenesis. RA also inhibits GSCs proliferation, invasion, lumen formation, and secretion of vascular endothelial growth factor, and reduces formation of angiogenic mimicry, thus promoting differentiation of GSCs and inhibiting their growth [37, 38]. High-dose RA treatment can efficiently inhibit solid tumor formation [39, 40]. Yet, contrary to expectations, RA did not have a beneficial effect in primary clinical trials for glioma patients [41, 42], and neither the combination of RA with temozolomide [43] nor radiotherapy [44] were successful [45, 46]. However, it was unclear whether the poor effect of RA treatment in glioma patients was associated with the RDH10 expression.

In this study, we analyzed the biological function of RDH10 in human gliomas. Analysis of GE-mini dataset demonstrated that the expression of RDH10 is increased in gliomas compared to normal brain tissues, especially in GBM [29]. In addition, high RDH10 expression was associated with malignant progression and poor prognosis in glioma patients, indicating that RDH10 may regulate human glioma development and progression. Our *in vitro* data demonstrated that RDH10 suppression inhibited glioma cell survival, proliferation, and invasion ability. *In vivo*, RDH10 suppression significantly reduced xenograft tumor growth. Together, our results demonstrate that RDH10 induces survival, proliferation, and invasion of glioma cells, both *in vivo* and *in vitro*.

The above results indicate that RDH10 is a potential oncogene in gliomas. To elucidate the mechanism as to how RDH10 promotes glioma cell proliferation and survival, we performed a whole-genome expression microarray in glioma cells with suppressed RDH10. As predicted, we found that RDH10 regulates a cluster of genes and signaling pathways related to cancer cell proliferation and survival. Based on the IPA database, the TWEAK signaling pathway ranked as the most inhibited pathway in RDH10-suppressed cells (Figures 5 and 6). The TWEAK/Fn14 signaling axis has been previously associated with tumor growth and metastasis, and therapeutic agents that target TWEAK or Fn14 are in development for use in cancer [47–49]. Previous studies have also shown that the TWEAK signaling plays a key role in glioma development and progression via the TWEAK–NF-κB axis [16, 50].

Our data demonstrate that RDH10 silencing suppresses TNFRSF12A (TWEAKR, Fn14), TNFSF12 (TWEAK), TRAF3, IKBKB (IKKβ), TGFBR1, and BMPR2 expression, while it increases TRAF1, MAP3K14 (NIK), NFKBIA (IκBα), NFKBIE (IκBε), TNFAIP3, GADD45A, and CDKN1A expression (Figures 6A–6C). These results are consistent with the previous studies indicating that the TWEAK–NF-κB axis is important for glioma development [16, 35]. Furthermore, we found that NF-κB activation blocked shRDH10-induced apoptosis and partially rescued impaired glioma cell proliferation of shRDH10-treated cells. Gene interaction network analysis and IPA analysis indicated that IκBα is in the center of this regulatory network; its downstream targets, including MMP family members, BCL2L1 (BCL-X) and BCL2L2 (BCL-W), were decreased (Figure 6D and 6E; Supplymentary Table 3). These results are consistent with previous studies [16, 35], and demonstrate that RDH10 promotes glioma progression through the TWEAK–NF-κB axis.

This is the first report that demonstrates the positive correlation between RDH10 expression and glioma progression and grades. Our results show that RDH10 knockdown inhibits glioma cell proliferation, survival, cell cycle, and invasion. In addition, microarray data demonstrate that RDH10 regulates multiple cancer-related genes and pathways, including the TWEAK –NF-κB axis. Together, our results indicate that RDH10 induces glioma development and progression, and suggest that it may serve as a potential novel target for human glioma treatment.

## MATERIALS AND METHODS

### Glioma samples and RDH10 immunohistochemistry (IHC)

We collected 150 tumor samples for our research, which was approved by the ethics committee of Beijing Tiantan Hospital, CMU; the different glioma grades were equally represented in the 150 samples. We used IHC to stain the formalin-fixed, paraffin-embedded tissues. Each slide was scored by the area and degree of positive staining. For statistical analysis, RDH10 staining was scored as follows: − (<5% positive nuclei) and + (6–100 % positive nuclei). We used SPSS17.0 to analyze the relationship between RDH10 expression and WHO gliomas grade. Statistical significance was calculated with Generalized Linear Model Regression (*P*<0.05).

### TCGA gene expression data

RDH10 expression in human glioma specimens was analyzed using publicly available TCGA Illumina RNA-Seq datasets from 667 glioma patients linked with their clinical parameters and follow-up information. We applied linear regression to study the gene expression and ordinal data analysis to analyze the association between RDH10 expression and WHO grades. We used Kaplan–Meier survival curves of the low- and high-risk groups separated by the level of RDH10 expression. Additionally, the log-rank test was applied to test the significance of differences in the survival curves between the two groups.

### Cell culture

Human glioma cell lines U87 and U251 were obtained from ATCC (Manassas, VA, USA). Cells were cultured in Dulbecco's modified Eagle's medium (Hyclone, Logan, UT, USA) supplemented using 10% fetal bovine serum (Hyclone, Logan, UT, USA) at 37°C in a humidified atmosphere containing 5% CO_2_.

### RDH10 shRNA design and lentiviral construction

We designed RDH10-shRNA sequences and constructed lentiviruses expressing these RDH10-shRNAs to inhibit RDH10 expression. The most effective RDH10-targeting shRNA had the target sequence: 5′-TACGATGCTGGAGATTAAT-3′and the sequence of Scr-shRNA was5′-TTCTCCGAACGTGTCACGT-3′. The pGCL-GFP-Lentivirus used to express shRNAs was purchased from Shanghai GenechemCo. Ltd. (Shanghai, China).

### RNA isolation and qPCR

Total RNA was extracted with TRIzol reagent (Invitrogen, Carlsbad, CA, USA). Approximately 2 μg of total RNA was reverse-transcribed into cDNA. PCR amplification was performed in triplicates with SYBR Master Mix (Takara, Shiga, Japan) using a Bio-Rad CFX96 qPCR detection system (Bio-Rad Laboratories, Hercules, CA, USA). Primer sequences are listed in Supplementary Table 3.

### Western blot analysis

We used standard techniques to perform western blotting, using GAPDH as a control for whole-cell lysates. Antibodies were the following: RDH10 (ab174340, Abcam, Cambridge, UK), CDKN1A (ab7960, Abcam), BIRC3 (ab32059, Abcam), BMPR2 (ab130206, Abcam), NFKBIA (ab7217, Abcam), TGFBR1 (ab31013, Abcam), GADD45A (ab180768, Abcam) and GAPDH (sc-32233, Santa Cruz Biotechnology, Dallas, TX, USA).

### Cell proliferation and analysis

After cells were treated with RDH10-shRNA or Scr-shRNA lentiviruses, they were incubated at 2000 cells per well in 96-well plates. We next used ArrayScan™ HCS (ThermoFisher, Waltham, MA, USA) to collect fluorescence images of each cell at 24h intervals for 5 d, and counted cell numbers in each well to create growth curves for each condition.

### MTT assay

We incubated cells seeded in 96-well plates at an initial density of 2000 cells per well. At each time point, 20 μL of MTT (5 mg/mL tetrazolium bromide, GE Healthcare, Little Chalfont, UK) was added into each well. After 4-h incubation, we added 150 μL of DMSO to solubilize the crystals for 20 min and the absorbance at 570 nm was recorded by an ELISA plate reader (Model 680, Bio-Rad Laboratories). For NF-κB activation, agonist fusicoccin was added 24 hours after cells were treated with RDH10-shRNA or Scr-shRNA lentivirus at a final concentration of 20 μM.

### Cell cycle analysis

Cell cycle profiles were examined using flow cytometry. First, cells treated with RDH10 -shRNA or Scr-shRNA were collected and fixed with cold 70 % alcohol for 30 min. Then, the cells were washed twice using PBS and dyed using propidium iodine, and then incubated with RNase in PBS. The suspension was filtered with a nylon mesh, and at least 1 × 10^5^ stained cells were analyzed by flow cytometry (Becton-Dickinson, Franklin Lakes, NJ, USA), and triplicate experiments were performed.

### Apoptosis analysis

Apoptosis was assayed by Annexin V-APC staining and detected by flow cytometry (Becton-Dickinson). For apoptosis analysis, cells were stained in 100 μL binding buffer containing 5 μL Annexin V-APC and 10μL propidium iodine (20 ng/mL), and then cultured in the dark at room temperature for 10–15 min. Apoptosis rates were measured by flow cytometry within 1 h.

### Cell invasion assay

Cells transfected with shRDH10 or shCtrl were collected, and a total of 1 × 10^5^ cells were seeded onto Corning® BioCoat™ Matrigel®™ Invasion Chambers (Corning, USA). Invasion of the cells through Matrigel to the underside was assessed 16 h (U87) and 40 h (U251) by staining with GIEMSA and counting cells under a microscope. The mean number of invaded cells per field was determined in nine fields per filter. Cells were counted under a light microscope. Assays were performed three times using triplicate wells.

### Microarray detection and data analysis

All microarray experiments were performed at Genechem (Shanghai, China) using RNA isolated from RDH10-shRNA and scr-shRNA U-87 cells. We used human GeneChip PrimeView (Affymetrix, Santa Clara, CA, USA) for microarray detection in accordance with the manufacturer's protocols. Arrays were scanned with a GeneChip Scanner 3000 (Affymetrix) to generate original data. We selected differentially expressed genes between RDH10-shRNA and Scr-shRNA cells using BH corrected *P* <0.05 and absolute FC>1.5. Pathway analysis, diseases and functions analysis, networks analysis and upstream genes analysis were performed by IPA.

### Animal experiments

Animal experiments were performed with 5-week-old BALB/c-A nude mice, which were purchased from Vital River Laboratories China. All procedures were conducted in accordance with the Chinese Council on Animal Care, and the protocol was approved by Beijing Tiantan Hospital, CMU. U87 cells (3 × 10^6^) were subcutaneously implanted into the right axillary region of nude mice to generate xenograft tumors. The mice were divided into two groups, RDH10-shRNA and Scr-shRNA (n=10). When the tumor size approached 100 mm^3^ (∼32 d), we measured tumor diameters with Vernier calipers every 48 h. Tumor volume was calculated by the following formula: v(mm^3^) = length×width^2^ ÷ 2. We harvested tumors for comprehensive analysis after 45 d.

### Statistical analysis

Statistical data were analyzed with the SPSS17.0 software package. All values were expressed as mean ± standard deviation (SD). The analysis of RDH10 expression in human tissue samples was performed using the survival analyses, generalized linear model, and Fisher's exact test. All experiments were repeated at least three times and statistical significance was considered at *P* < 0.05.

## SUPPLEMENTARY MATERIALS TABLES



## Abbreviations

(CMU): Capital Medical University
(FACS): Fluorescence activated Cell Sorting
(FC): fold change
(GBM): glioblastoma
(GTEx): Genotype-Tissue Expression
(GSCs): glioma stem-like cells
(HCS): High Content Screening
(HGG): high-grade gliomas
(IPA): Ingenuity Pathway Analysis
(LGG): low-grade gliomas
(TCGA): The Cancer Genome Atlas
(WHO): World Health Organization
