# Supplementary material for: Retinol dehydrogenase-10 promotes development and progression of human glioma via the TWEAK-NF-κB axis
Source: Oncotarget. 2017 Oct 27;8(62):105262–75. doi: 10.18632/oncotarget.22166 (PMC5739636; doi:10.18632/oncotarget.22166)
Supplement: Supplementary file 1 [file oncotarget-08-105262-s001.pdf]

Retinol dehydrogenase-10 promotes development and progression of human glioma via the TWEAK-NF-κB axis

SUPPLYMENTARY MATERIALS

Supplymentary Table 1: Expression of RDH10 in LGG and GBM samples in TCGA

		Level of expression of the gene		Total	P value
		Low expression	High expression		
RDH10 expression	LGG	329	186	515	.000
	GBM	5	147	152	
Total		334	333	667	

Supplymentary Table 2: List of IKKα (NFKBIA) downstream genes expression changed in microarray

Genes	Entrez Gene Name	FC (shRDH10/shCtrl)	Corrected P-Value
BIRC3	baculoviral IAP repeat containing 3	3.37	3.96E-04
BCL2L1	BCL2 like 1	-1.66	1.76E-03
BCL2L2	BCL2 like 2	-1.54	9.91E-05
CDKN1A	cyclin dependent kinase inhibitor 1A	1.82	8.08E-05
DDIT3	DNA damage inducible transcript 3	1.59	3.43E-04
GADD45A	growth arrest and DNA damage inducible alpha	1.98	3.71E-05
GADD45B	growth arrest and DNA damage inducible beta	1.93	6.99E-05
MMP1	matrix metallopeptidase 1	-2.25	9.28E-05
MMP2	matrix metallopeptidase 2	-2.20	5.82604E-05
MMP3	matrix metallopeptidase 3	-2.05	3.87E-05
MMP7	matrix metallopeptidase 7	-2.09	8.84E-04
RAC1	ras-related C3 botulinum toxin substrate 1 (rho family, small GTP binding protein Rac1)	-2.33	7.18E-05
YAP1	Yes associated protein 1	-1.59	2.23E-04

Supplymentary Table 3: List of qPCR primers

name	sequences
**Primers for qPCR:**	
RDH10 sense:	5'- TGGGACATCAACACGCAAAGC-3′
RDH10 antisense:	5'- TGCAAGTTACAGTGGGGCAGA-3′
TNFRSF12A sence:	5'- CTCTGAGCCTGACCTTCGTG -3′
TNFRSF12A antisence:	5'- TCTCCGCCGGTCTCCTCTAT -3′
TRAF3 sence:	5'- GCGTGTCAAGAGAGCATCGTT-3′
TRAF3 antisence:	5'- GCAGATGTCCCAGCATTAACT-3′
IKBKB sence	5'- GAACGGATGATGGCTCTG-3′
IKBKB antisence	5'- CCCTTGCTTGCTCCTCTA-3′
TGFBR1 sence	5'- ACGGCGTTACAGTGTTTCTG -3′
TGFBR1 antisence	5'- GCACATACAAACGGCCTATCTC -3′
BMPR2 sence	5'- ACGGGTATCTTTTGTTGGTGT-3′
BMPR2 antisence	5'- TGTCAGCTTTCATAGTGGCATC-3′
TRAF1 sence	5'- CATCTGTCGCTCTTCATCGT -3
TRAF1 antisence	5'- AAGTGCTGGTCTCCACAATG -3
MAP3K14 sence	5'- AAAATGGCCCGTGTGTGTTG -3
MAP3K14 antisence	5'- GCCGAGTGGAGACTCATCC -3
NFKBIA sence	5'- CCTGAAGGCTACCAACTACA -3
NFKBIA antisence	5'- ATCAGCACCCAAGGACAC -3
NFKBIE sence	5'- TTACCCATGTTGGGTCAGCC -3
NFKBIE antisence	5'- AACGGTGTTTCAGGGTCCTC -3
TNFAIP3 sence	5'- CCACGATGCTCAGGTTTG -3
TNFAIP3 antisence	5'- TCCCTTTCTCAGCCAAGAC -3
GADD45A sence	5'- GAGAGCAGAAGACCGAAAGG -3
GADD45A antisence	5'- CAGCAGGCACAACACCAC -3
CDKN1A sence	5'- TAGGCGGTTGAATGAGAGG -3
CDKN1A antisence	5'- GTGACAGCGATGGGAAGG -3
GAPDH sence	5'- TGACTTCAACAGCGACACCCA -3
GAPDH antisence	5'- CACCCTGTTGCTGTAGCCAAA -3
